# Heterochromatin Protein 1 (HP1a) Positively Regulates Euchromatic Gene Expression through RNA Transcript Association and Interaction with hnRNPs in *Drosophila*

**DOI:** 10.1371/journal.pgen.1000670

**Published:** 2009-10-02

**Authors:** Lucia Piacentini, Laura Fanti, Rodolfo Negri, Valerio Del Vescovo, Alessandro Fatica, Fabio Altieri, Sergio Pimpinelli

**Affiliations:** 1 Istituto Pasteur, Fondazione Cenci Bolognetti and Dipartimento di Genetica e Biologia Molecolare, Università La Sapienza, Roma, Italy; 2 Istituto Pasteur, Fondazione Cenci Bolognetti and Dipartimento di Biologia Cellulare e Dello Sviluppo, Università La Sapienza, Roma, Italy; 3 Istituto Pasteur, Fondazione Cenci Bolognetti and Dipartimento di Scienze Biochimiche A. Rossi Fanelli, Università La Sapienza, Roma, Italy; Fred Hutchinson Cancer Research Center, United States of America

## Abstract

Heterochromatin Protein 1 (HP1a) is a well-known conserved protein involved in heterochromatin formation and gene silencing in different species including humans. A general model has been proposed for heterochromatin formation and epigenetic gene silencing in different species that implies an essential role for HP1a. According to the model, histone methyltransferase enzymes (HMTases) methylate the histone H3 at lysine 9 (H3K9me), creating selective binding sites for itself and the chromodomain of HP1a. This complex is thought to form a higher order chromatin state that represses gene activity. It has also been found that HP1a plays a role in telomere capping. Surprisingly, recent studies have shown that HP1a is present at many euchromatic sites along polytene chromosomes of *Drosophila melanogaster*, including the developmental and heat-shock-induced puffs, and that this protein can be removed from these sites by in vivo RNase treatment, thus suggesting an association of HP1a with the transcripts of many active genes. To test this suggestion, we performed an extensive screening by RIP-chip assay (RNA–immunoprecipitation on microarrays), and we found that HP1a is associated with transcripts of more than one hundred euchromatic genes. An expression analysis in HP1a mutants shows that HP1a is required for positive regulation of these genes. Cytogenetic and molecular assays show that HP1a also interacts with the well known proteins DDP1, HRB87F, and PEP, which belong to different classes of heterogeneous nuclear ribonucleoproteins (hnRNPs) involved in RNA processing. Surprisingly, we found that all these hnRNP proteins also bind heterochromatin and are dominant suppressors of position effect variegation. Together, our data show novel and unexpected functions for HP1a and hnRNPs proteins. All these proteins are in fact involved both in RNA transcript processing and in heterochromatin formation. This suggests that, in general, similar epigenetic mechanisms have a significant role on both RNA and heterochromatin metabolisms.

## Author Summary

Heterochromatin Protein 1 (HP1a) is a very well known prototype protein of a general model for heterochromatin formation and epigenetic gene silencing in different species including humans. Here, we report our experiments showing that HP1a is also required for the positive regulation of more than one hundred euchromatic genes by its association with the corresponding RNA transcripts and by its interaction with heterogeneous nuclear ribonucleoproteins (hnRNPs) belonging to different classes. Importantly, we also found that all the tested hnRNP proteins bind to the heterochromatin and are dominant suppressors of position effect variegation, thus suggesting they also have a role in heterochromatin organization. Taken together, our data show novel and important functions, not only for HP1a, but also for hnRNPs, which were previously believed to participate only in RNA processing. These results shed new light on the epigenetic mechanisms of gene silencing and gene expression. They also establish a link between RNA transcript metabolism and heterochromatin formation and change several aspects of the canonical views about these apparently different processes.

## Introduction

HP1a isoform is the original chromosomal protein first discovered in *Drosophila melanogaster* through its association with the heterochromatin [1],[2]. Molecular studies have shown that HP1a is a phylogenetically highly conserved protein [3]–[5] with two prominent structural motifs, the chromo domain [6] and chromoshadow domain [7], important for chromatin binding and protein interactions respectively. In *Drosophila*, HP1a is encoded by the *Su(var)2–5* locus, a dosage-dependent modifier of position effect variegation (PEV) [8]. Both the heterochromatic location of HP1a and its effect on PEV demonstrate its essential role in heterochromatin formation. Different sets of data have established the ability of HP1a to associate with several different proteins [9]–[11]. A general model has been proposed for heterochromatin formation and epigenetic gene silencing in different species. According to the model, histone methyltransferase enzymes (HMTases) methylate the histone H3 at lysine 9 (H3K9me), creating selective binding sites for themselves and for the chromodomain of HP1a [12]. This complex is thought to form a higher order chromatin state that represses gene activity.

In addition to being required for heterochromatin formation, HP1a also plays a critical role in telomere capping and the telomere transcriptional repression in *Drosophila* [13]–[15]. HP1a is a structural component of all *Drosophila* telomeres, and its absence results in extensive telomeric fusions and hypertranscription of telomeric sequences.

A detailed cytological analysis of the distribution of HP1a in polytene chromosomes of *Drosophila melanogaster* using an anti-HP1a antibody has demonstrated the presence of the protein at about 190 euchromatic sites, including the developmental and heat-shock induced puffs [16]. Intriguingly, when the heat-shock induced expression of the HSP70-encoding gene was examined in larvae either lacking or with a superabundance of HP1a, HP1a was shown to be positively involved in *Hsp70* gene activity [17]. Other recent experiments also support a positive role of HP1a in gene expression. Many euchromatic genes in *Drosophila* are down-regulated in HP1a deficient larvae. Although it is still unknown how many of these genes are direct targets of HP1a, the same study showed that HP1a is associated with some of them [18]. Similar results were seen when HP1a was depleted in cultured cells [19]. High-resolution mapping experiments have also shown that HP1a is associated with transcriptionally active chromatin in *Drosophila* [20]. Also relevant is the prior observation that, in *Drosophila*, the *in vivo* RNase treatment of polytene chromosomes of wild type larvae removes almost all the euchromatic HP1a immunosignals. This implies that HP1a directly associates with the transcripts of many active genes [17].

Together, these data lead to the hypothesis that HP1a is involved in the positive regulation of gene expression by binding RNA transcripts. We describe here our experiments to test this hypothesis. Our results show that HP1a can directly bind RNA *in vivo* and that it interacts with the active RNA-polymerase II (Pol II). Most importantly, a “RIP-Chip” (RNA-immunoprecipitation on microarrays) analysis shows that HP1a associates with the RNA transcripts of more than one hundred genes. We found that HP1a positively regulates the expression of these genes by also interacting with DDP1, HRB87F and PEP, which belong to different classes of heterogeneous nuclear ribonucleoproteins (hnRNPs) involved in RNA processing. Surprisingly, we found that all these hnRNP proteins also bind heterochromatin and are dominant suppressors of position effect variegation.

## Results

### HP1a interacts with active Pol II and directly binds RNA transcripts

**Figure 1:**
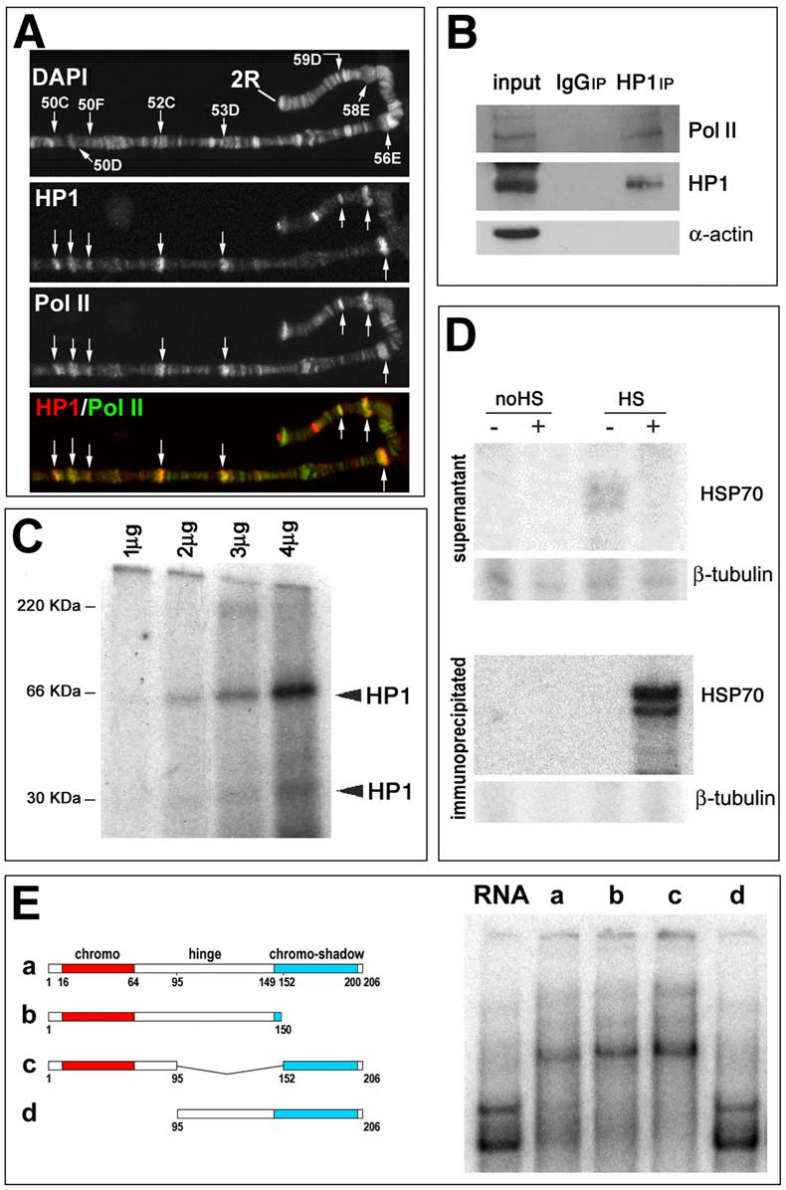
HP1a can directly bind RNA in vitro and in vivo and interacts with Pol II. (A) Immunolocalization of HP1a and active Pol II on polytene chromosomes of *D. melanogaster*. Signals produced by the two antibodies show an extensive colocalization. (B) Coimmunoprecipitation of HP1a and Pol II by an anti-HP1a antibody. To test the specificity of HP1a with Pol II interaction, we also probed with an antibody against α-actin. (C) UV crosslinking after incubation of four different concentrations of HP1a with Hsp70 RNA *in vitro*. Note that radioactive HP1a bands with molecular weights corresponding to an HP1a dimer and monomer are present in the third and fourth lanes. (D) Primer extension of RNA immunoprecipitated from S2 cells with CIA9 antibody. Two signals are present only in HP1a immunoprecipitates of heat-shocked cells (HS). (E) Electromobility shift assay. Left, a diagram of the HP1a fragments used in the gel shift assay. Right, the results of EMSA of radiolabelled HSP70 RNA using the different HP1a fragments (50 ng). The absence of shift (lane d) using the HP1a fragment lacking the chromo-domain strongly suggests that this part of the protein is responsible for the binding of HP1a to the RNA transcripts.

The binding of HP1a to heat-shock induced puffs and to the majority of euchromatic loci seems to be mediated by the presence of nascent RNA, since RNase treatment removes euchromatic HP1a immunosignals [17]. To test if the multiple euchromatic HP1a binding sites correspond to active genes, we immunostained the polytene chromosomes of salivary glands with a specific antibody directed against HP1a, and an antibody against the active form of Pol II Phospho Ser2. As shown in Figure 1A and Figure S2A, HP1a and Pol II have an extensive co-localization. These results are confirmed by immunoprecipitation experiments using the anti-HP1a antibody. A western blot of the immunoprecipitated proteins reveals the presence of Pol II (Figure 1B).

We then verified a direct interaction of HP1a with RNA transcripts through *in vitro* and *in vivo* experiments. Recombinant HP1a was incubated with Hsp70 RNA which had been previously transcribed and marked radioactively. The protein HP1a was then cross-linked to the RNA by UV and resolved by SDS-PAGE. Radioactive signals at the positions corresponding to the molecular weight of the HP1a monomer and dimer show that the protein is able to bind Hsp70 RNA *in vitro* (Figure 1C). The ability of HP1a to interact with the Hsp70 RNA *in vivo* was analyzed by primer extension on the population of RNA immunoprecipitated from Schneider's (S2) cultured cells with the monoclonal C1A9 (anti-HP1a) antibody. As shown in Figure 1D, Hsp70 RNA is present only in the immunoprecipitated RNA from heat-shocked cells, confirming the ability of HP1a to bind Hsp70 RNA *in vivo*. Both these results indicate the association of HP1a with transcriptionally active genomic regions. To determine which part of the protein is responsible for its RNA binding, we performed a gel shift assay on the series of HP1a fragments indicated in Figure 1E [15],[21],[22]. As Figure 1E shows, we found that only the HP1a fragments containing the chromodomain are capable of producing a gel shift of RNA. These results strongly suggest that the chromodomain region is required for the direct binding of HP1a to RNA transcripts.

### Identification of the target transcripts of HP1a by “RIP-Chip” (RNA-immunoprecipitation on microarrays) analysis

We used an optimized “RIP-Chip” (RNA-immunoprecipitation on microarrays) protocol to screen for the target RNA transcripts of HP1a. After the advent of RIP-chip technologies, several related methods were developed using physical or chemical RNA-protein cross-linking to identify the multiple RNA targets of RNA binding protein [23]. We decided not to use cross-linking in our S2 cell extracts to avoid any artifacts produced by cross-linking reagents, such as the reduction of cell lysis, the introduction of sequence bias, an increase in background and a less-than-complete reversibility. Instead, we optimized the RIP-Chip conditions to find *in vivo* the RNA substrates of HP1a protein while minimizing possible artifacts. Control experiments were done to ensure the absolute requirements for productive amplification and labeling in the RT reaction and to test for the presence of HP1a in the immunoprecipitated RNA (not shown). After background subtraction, the net intensity for each spot and the ratio between the IP and input were calculated. Four independent experiments were performed, and after data filtering to exclude artifacts and low-signal spots, the subsequent analysis was carried out on the 5780 cDNA clones which gave reliable signals in at least 3 out of 4 experiments. For each experiment, data were ordered by ranking, and the median rank was calculated for each cDNA clone (Table S1). Table S2 shows the median percentile rank (MPR) between IP and input for the cDNA clones within the 90^th^ percentile. The same table reports the results of two independent experiments done with the identical procedure but without the antibody against HP1a. It is evident that most of the putative HP1a targets show a much lower percentile rank in these control experiments, confirming the specificity of the immunoprecipitation.

We identified 105 genuine transcript targets, reported in Table S3. These transcripts correspond to genes that, constitutively, support a very active rate of transcription. To test whether these genes correspond to the most abundant transcripts or are specifically associated with HP1a, we identified the 200 most abundant transcripts in the input RNA and analysed their median percentile rank in HP1a IP. Only 6/200 transcripts are included in the list of the best HP1a binders (IP rank≥0.9) (Table S4). Moreover, the median IP rank of the 200 most abundant transcripts is 0.45, while their median rank in the mock experiments is 0.61. Thus, if there is a bias in the HP1a experiments, it is toward less abundant, rather than more abundant transcripts. The signal intensity median rank for the best HP1a binders (IP rank≥0.9) is 0.56, indicating that the RNAs bound by HP1a tend to be only slightly more abundant than average.

De Wit et al. (2007) did a high resolution analysis of HP1a binding on *Drosophila* chromosomes 2 and 4 using the DNA adenine methyltransferase identification (DamID) technique [20]. This technique is used to make genome-wide maps of DNA-interacting proteins. DamID consists in making a fusion protein composed of the protein of interest and DNA adenine methyltransferase (Dam). Expression of this protein permits the methylation of adenines around the sites of the protein-DNA binding. The methylated sequences are amplified and identified by hybridization to microarrays. The authors identified 357 genes, with an average HP1a-Dam methylase vs Dam methylase log2-ratio along the entire gene >1, out of 3992 genes represented on the microarray (8.9%). A total of 189 genes (4.7%) showed an average log2 ratio >2, considered a more stringent cut-off by the authors. The pattern of increased methylation typically spanned the entire gene coding regions. We looked at the average HP1a-Dam/Dam log2 ratio of 21 genes present on chromosome 2 which showed >0.9 MP Rank in our RNA binding experiments. No gene showed an average Log2 ratio >1 (Table S5); the highest average ratio was 0.59. Moreover, only 6/21 genes (29%) showed significant localized clusters of HP1a-Dam/Dam log2 values >0.5 (Figure S1). These data suggest that when HP1a-Dam methylase fusion protein is bound via RNA, it retains a limited capacity to extensively methylate the DNA gene coding region. This limitation could be qualitative and/or quantitative and could be due to several factors: the methylating domain could be masked by interactions with RNA; the methylating domain could be masked by protein partners which are exclusively present when HP1a is bound to RNA; methylation could be less efficient due to a peculiar chromatin structure selectively present on HP1a RNA targets. We think that these observations should be taken in account when trying to reconstruct the complex pattern of interaction of this pleiotropic protein.

**Figure 2:**
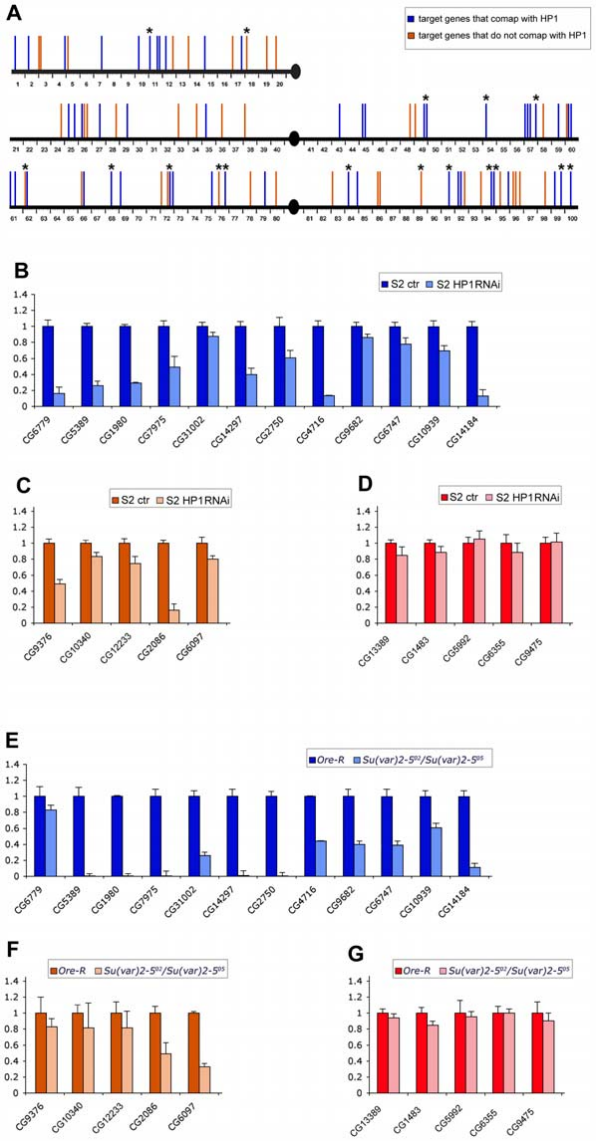
The genes corresponding to transcript targets of HP1a in S2 cells show a good overlap with HP1a binding sites on polytene chromosomes and appear down-regulated in both S2 cells lacking HP1a and HP1a mutant larvae. (A) Localization of HP1a binding sites and the genes corresponding to the HP1a target transcripts along polytene chromosomes of *Drosophila* wild type larvae. Blue bars represent sites where the HP1a target genes overlap with HP1a immunosignals; orange bars indicate the localization of HP1a target genes that do not overlap with HP1a immunosignals. (B) Quantitative RT–PCR analysis of the expression, in S2 cells treated with dsRNA of HP1a, of a sub-set of target genes that overlap with HP1a on polytene chromosomes whose position is indicated in (A) by blue bars marked with asterisks and (C) a sub-set of HP1a target genes that do not overlap with HP1a on polytene chromosomes whose position is indicated in (A) by orange bars marked with asterisks. (D) Quantitative RT-PCR analysis of the expression, in S2 cells treated with dsRNA of HP1a, of a sub-set of genes that were not found among the HP1a target genes in S2 cells and do not co-map with any of the HP1a immunosignals along the polytene chromosomes. (E) Quantitative RT-PCR analysis of the expression, in wild type and HP1a mutant larvae, of the same sub-set of target genes reported in Figure 2B. (F) Quantitative RT-PCR analysis of the expression, in wild type and HP1a mutant larvae, of the same sub-set of target genes reported in Figure 2C. (G) Quantitative RT-PCR analysis of the expression, in wild type and HP1a mutant larvae, of the same sub-set of non target genes reported in Figure 2D.

When we compare the HP1a euchromatic binding sites and the location of the genes corresponding to the RNA target sites in polytene chromosomes we find that about half of the selected targets (52/105) map in the same cytological band or in bands immediately adjacent to where HP1a has been located [16] (Figure 2A) (see also Table S3). This correlation appears excellent taking into account that the target transcripts were identified in cultured cells while the cytological immunosignals came from polytene chromosomes of larval salivary glands.

### HP1a positively regulates gene expression by binding RNA transcripts

**Figure 3:**
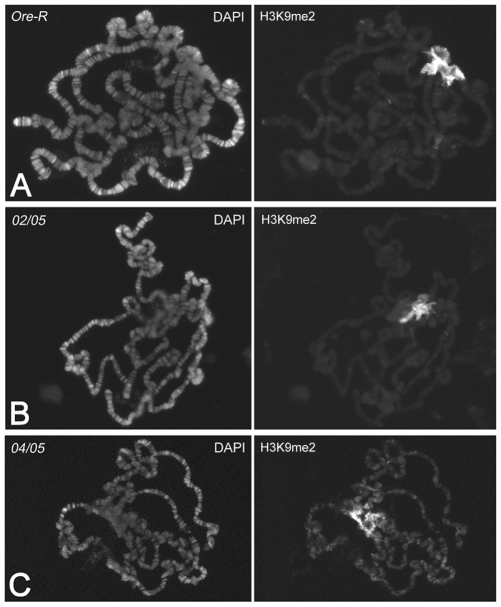
A mutated chromodomain of HP1a does not modify the immunopattern of Histone H3 di-methylated at lysine 9 (H3K9me2). (A) Immunopattern of H3K9me2 on polytene chromosomes of wild type larvae (*Ore-R*). The immunosignals are mainly present on the chromocenter. (B) H3K9me2 immunopattern on polytene chromosomes of *Su(var)2–5^02^/Su(var)2–5^05^* (*02/05*) larvae. The immunopattern is very similar to that of wild type. (C) H3K9me2 immunopattern on polytene chromosomes of *Su(var)2–5^04^/Su(var)2–5^05^* (*04/05*) null mutant larvae. In this case, the H3K9me2 immunosignals have been redistributed, even on euchromatic regions.

To see if HP1a upregulates gene expression by binding to RNA, we did a real time RT-PCR analysis of the expression of 17 genes corresponding to the HP1a target transcripts, in S2 cells treated with dsRNA for HP1a and in *Su(var)2–5^02^/Su(var)2–5^05^* larvae, which express an HP1a with a functionally inactive chromodomain. We chose 12 genes that comap with HP1a immunosignals on polytene chromosomes and five others located in regions apparently devoid of HP1a immunosignals (Figure 2A). As a negative control, we tested five genes that do not comap with HP1a in salivary glands and whose transcripts were not HP1a targets in S2 cells. As shown in Figure 2B and 2C, we found a significant reduction in all target transcripts of HP1a while we did not observe any effects on the amount of non-target transcripts (Figure 2D). In HP1a mutant larvae, we found a significant reduction in transcripts of the 12 genes that comap with HP1a (Figure 2E). Three of the genes that do not overlap with any HP1a signals did not show any significant variation between mutant and wild type larvae (Figure 2F). Therefore, these genes do not seem to be regulated by HP1a in larval cells. For the other two genes which do not comap with HP1a on polytene chromosomes, we observed a reduction in transcripts as in S2 cells, probably due to a down-regulation of their expression in other larval tissues; it is possible, in fact, that HP1a binds these transcripts in other larval tissues rather than in salivary glands. As observed in S2 cells, the results of RT-PCR analysis in HP1a mutant larvae clearly show no effect on the amount of the non HP1a target transcripts (Figure 2G). This also implies that the lack of HP1a induces a specific effect in gene expression and not a general effect in gene expression due to a larval lethality induced by the mutation. Previous observations have shown a spreading of H3K9 methylation in salivary glands of HP1a null mutant larvae [22], suggesting a general effect on gene transcription following the complete loss of HP1a. To test this possibility, we analyzed the H3K9 methylation along the polytene chromosomes in *Su(var)2–5^02^/Su(var)2–5^05^* (*02/05*) mutants compared to the wild type and to the null *Su(var)2–5^04^/Su(var)2–5^05^* (*04/05*) mutants. While in the null mutants a H3K9me2 euchromatic redistribution is evident (Figure 3C), in the *Su(var)2–5^02^* mutants (Figure 3B) the pattern is similar to that of wild type (Figure 3A), with no spreading of H3K9 methylation. This strongly supports the view that the *Su(var)2–5^02^* mutation affects the amount of transcripts of specific genes.

### HP1a interacts with hnRNP proteins

**Figure 4:**
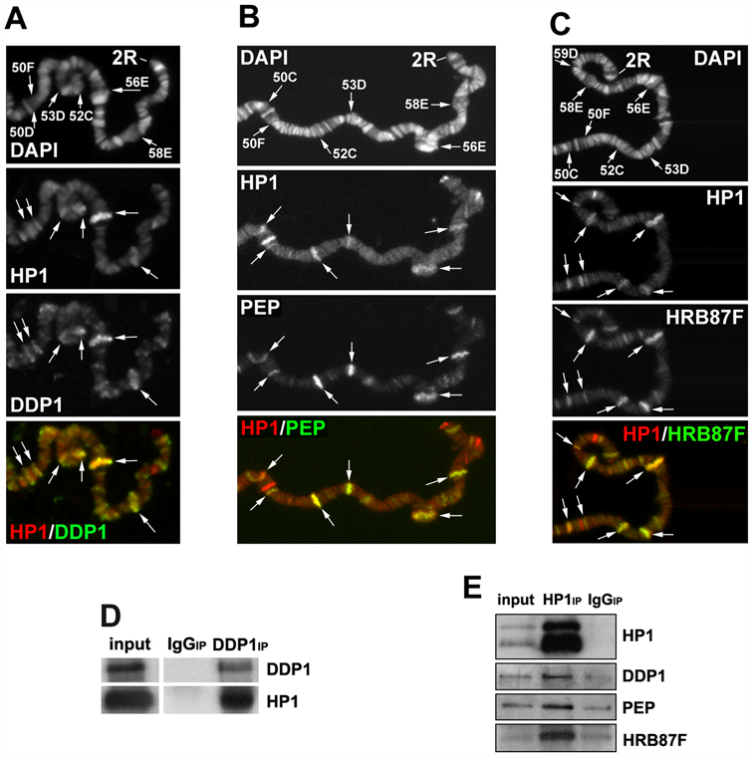
HP1a associates with and colocalizes on polytene chromosomes with DDP1, HRB87F, and PEP hnRNP proteins. (A–C) A part of the right arm of a wild type polytene second chromosome simulteneously immunostained with the anti-HP1a antibody and an antibody against: (A) DDP1, (B) PEP, and (C) HRB87F. There is an extensive colocalization of HP1a with each of the other proteins (arrows). We could not perform simultaneous immunostaining among the DDP1, PEP and HRB87F because the available specific antibodies were made in mouse. However, the colocalization of each protein with HP1a in same regions indicated that all the proteins colocalize in such regions. (For simultaneous immunostainings on whole polytene chromosomes, see Figure S3). (D) Coimmunoprecipitation of HP1a with DDP1 by an anti-DDP1 antibody. (E) Coimmunoprecipitation of HP1a with DDP1, PEP, and HRB87F proteins by the C1A9 anti-HP1a antibody.

The direct association of HP1a with RNA transcripts suggests that HP1a could be part of one or more ribonucleic complexes. Previous data have shown an interaction between HP1a and DDP1, a multi-KH-domain vigilin that binds single-stranded nucleic acids with high affinity *in vitro* [24],[25]. The KH-domain is a motif identified for the first time in the human heterogeneous nuclear ribonucleoprotein K (hnRNP K) [26]. In *Drosophila*, HP1a coimmunoprecipitates (Figure 4D) and extensively colocalizes with DDP1 protein on sub-regions of the heterochromatin and in many sites along the euchromatic arms of polytene chromosomes [24] (see also Figure 4A and Figure S2B). In fact, the binding of HP1a to heterochromatin seems to depend on DDP1 [25]. Treatment with RNase abolishes the DDP1 euchromatic immunopatterns but does not significantly affect the heterochromatin localization (data not shown). It may be that DDP1 binds single-stranded DNA in the chromocenter and RNA transcripts along the euchromatic arms. We asked whether HP1a might also interact with the hnRNP proteins HRB87F and PEP. Both these proteins associate with Hrb57A, another hnRNP protein that, like DDP1, is closely related to the human hnRNP K [27]. Both these proteins, like HP1a, colocalize with the active form of Pol II along polytene chromosomes [28]. HRB87F is the closest *Drosophila* homolog to mammalian A/B type hnRNP [29], which can bind both RNA and single-stranded DNA [30]. Like HP1a [17], HRB87F undergoes a dramatic chromosomal redistribution after heat-shock [31]. PEP (Peptide on Ecdysone Puffs) is a unique zinc finger protein that, like HP1a, is found preferentially associated on ecdysone-induced puffs as well as other regions of polytene chromosomes [32]. It has been shown that PEP can bind DNA and, with higher affinity, RNA [33].

A comparison of the HP1a immunopattern on polytene chromosomes with those produced by specific antibodies against PEP (Figure 4B and Figure S2C) and HRB87F (Figure 4C and Figure S2D) demonstrates that HP1a colocalizes with both these proteins along the euchromatic arms and partially on the heterochromatic chromocenter. We made an approximate estimation of the extent of colocalization by counting the number of overlapping sites; about 70% of HP1a signals overlap with those of both proteins. There are also few sites where HP1a overlaps only with one or the other protein. In immunoprecipitation experiments using the anti-HP1a C1A9 antibody, HP1a coprecipitates with both HRB87F and PEP proteins and DDP1 (Figure 4E).

**Figure 5:**
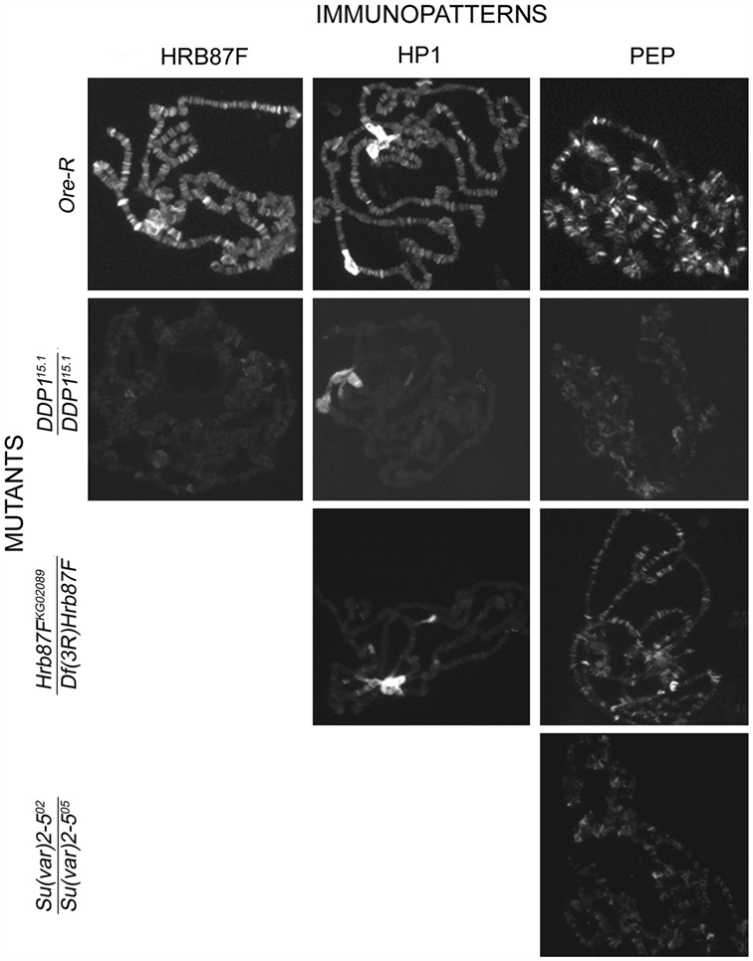
Hierarchical dependence of HP1a, DDP1, HRB87F, and PEP in their assembly on RNA transcripts. Immunopatterns of each protein on polytene chromosomes of wild type larvae and larvae mutant for the genes encoding each of the other proteins. (Only the abnormal patterns are reported here; a complete version of the results is in Figure S3). In DDP1 mutants, the immunopatterns of the other proteins are abnormal. In *Hrb87F* mutants, the HP1a and PEP immunopatterns are abnormal while the DDP1 immunopattern is unaltered. In HP1a mutants only the PEP immunopattern is abnormal, whereas in PEP mutants the immunopatterns of all other proteins are normal (see Figure S3).

**Figure 6:**
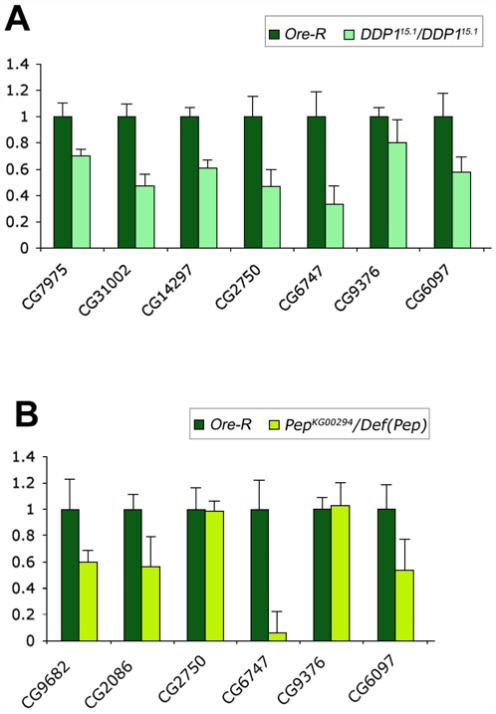
Genes corresponding to transcript targets of HP1a in S2 cells appear down-regulated also in DDP1 and PEP mutants larvae. (A) Quantitative RT–PCR analysis of the expression in wild type *Ore-R* and DDP1 mutant larvae showing a significant decrease in the amount of the transcripts corresponding to all genes. (B) Quantitative RT–PCR analysis in wild type *Ore-R* and PEP mutant larvae. In this case a significant decrease in the amount of the transcripts is evident for four of the six analyzed genes.

To analyze possible interdependencies in chromosomal localization among all these proteins we immunolocated each protein on the polytene chromosomes of larvae mutant for genes encoding each of the other proteins. From the results reported in Figure 5 and in Figure S3, it appears that the correct localization of each protein on the euchromatin depends on the presence of the others according to a hierarchical order with DDP1 on the top: DDP1>HRB87F>HP1a>PEP. We conclude that all these proteins interact for their localization in an ordered manner. Intriguingly, we have also observed that DDP1 is required for the localization of HRB87F and PEP proteins on the chromocenter while DDP1 and Hrb87F mutations partially affect the HP1a heterochromatic immunopattern. We further tested the functional interaction of HP1a with DDP1 and PEP by analyzing the expression of a subset of genes, corresponding to the HP1a target transcripts, in DDP1 and PEP mutant larvae. As reported in Figure 6, we found similar effects to those observed on the expression of same genes in HP1a mutant larvae.

To test whether HP1a forms a nuclear complex with the interacting hnRNPs, *Drosophila* S2 cells were transfected with a plasmid coding for FLAG-tagged HP1a under the control of actin5C promoter. As a control, cells were transfected with the same plasmid coding for FLAG-tagged GFP protein. The efficiency of transfection was monitored by comparing microscopically the amount of fluorescence in control vs experimental cells, and we found it was generally 65%–70%.

**Figure 7:**
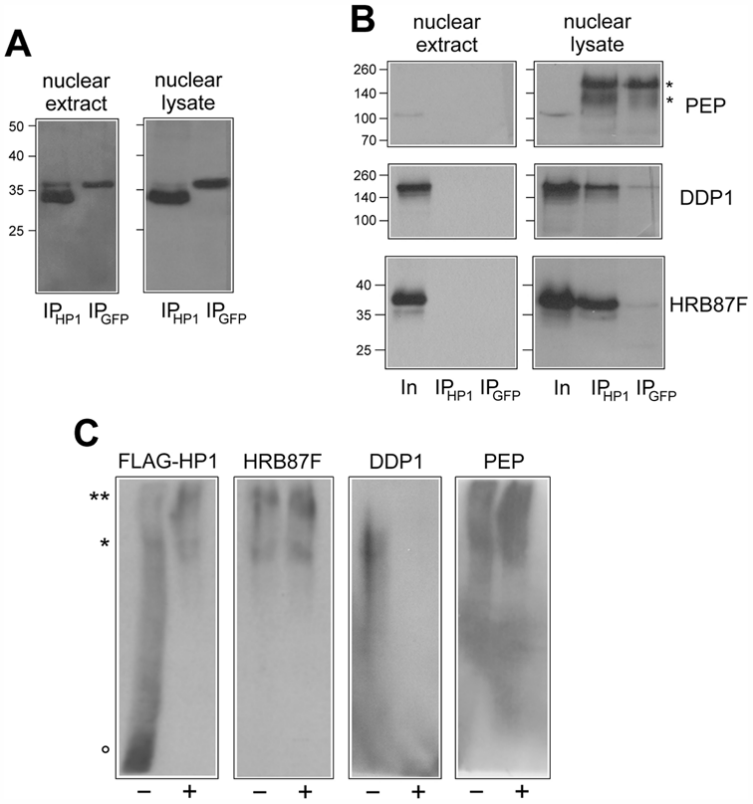
HP1a seems to be involved in forming a complex with hnRNP proteins. (A) Western blot analysis with anti-FLAG antibody of immunopurified fractions obtained from nuclear extracts and nuclear lysates of S2 cells tranfected with FLAG-tagged HP1a (IP_HP1_) or FLAG-tagged GFP (IP_GFP_). Aliquots corresponding to 3×10^6^ cells processed for immunopurification are loaded on each lane. Molecular weight markers, expressed in kDalton, are reported on the left. (B) Western blot analysis with anti-PEP, anti-DDP1 and anti-HRB87F antibodies of immunopurified fractions obtained from nuclear extracts and nuclear lysates of S2 cells tranfected with FLAG-tagged HP1a (IP_HP1_) or FLAG-tagged GFP (IP_GFP_). Aliquots corresponding to nuclear extracts and lysates obtained from 3×10^5^ cells before immunopurification (lanes In) are compared with aliquots corresponding to 3×10^6^ cells processed for immunopurification (lanes IP_HP1_ and IP_GFP_). Molecular weight markers, expressed in kDalton, are reported on the left. Bands marked with asterisks correspond to mouse IgGs present in the sample and revealed by secondary antibodies. (C) Western blot analysis with anti-FLAG, anti-HRB87F, anti-DDP1 and anti-PEP antibodies of immunopurified fractions obtained from nuclear lysates of S2 cells tranfected with FLAG-tagged HP1a resolved by native-PAGE before (lanes −) and after (lanes +) RNAse treatment. Aliquots corresponding to 3×10^6^ cells processed for immunopurification are loaded on each lane. Complexes with low-mobility (** and *) and a fast mobility form of HP1a (°) are indicated.

To concentrate solely on nuclear HP1a, transfected cells were lysed by hypotonic buffer and the nuclei were processed for immunopurification. Immunopurification was done on either soluble nuclear extract, corresponding to the nucleoplasmic fraction, or total nuclear lysate, corresponding to a soluble chromatin fraction. Both fractions, obtained from HP1a and GFP transfected cells, were immunopurified using anti-FLAG monoclonal antibodies and protein G-coated magnetic beads. Immunoadsorbed fractions were specifically eluted with a 3X-FLAG peptide, resolved by electrophoresis and analysed by western blot. Figure 7A reports the western blot analysis of nuclear extracts and lysates obtained from HP1a and GFP transfected cells using anti-FLAG antibody. A band corresponding to FLAG-tagged protein is clearly evident in both immunopurified fractions, confirming the efficiency of transfection and immunopurification. Figure 7B reports the western blot analysis of nuclear extracts and lysates obtained from HP1a and GFP transfected cells using anti-PEP, anti-DDP1 and anti-HRB87F antibodies. It should be noted that the nuclear fractions after immunopurification (Ip lane) represent 10X the number of cells as those of the nuclear fractions before immunopurification (Input lane). Analysis of the nuclear lysates shows that all three analyzed proteins coimmunopurify with HP1a (IP_HP1a_ lanes) but are absent in the control immunopurification (IP_GFP_ lanes), confirming their interaction with HP1a. Since the same proteins are absent in the immunopurified fraction obtained from nuclear extracts (the soluble nucleoplasmic fraction) we hypothesize that these HP1a-containing complexes are mainly associated with chromatin.

To confirm the association of DDP1, PEP and HRB87F in HP1a-containing complexes, immunopurified fractions were resolved by native-PAGE followed by western blot analysis.

Figure 7C shows that HP1a is involved in nuclear complexes with different mobility which include all three protein tested (DDP1, PEP and HRB87F). Two low-mobility complexes are evident: the first one of HP1a, PEP and HRB87FT (marked with **) and the second one of all the proteins tested (marked with *). A fast mobility form of HP1a is also evident which does not comigrate with any of the other proteins (marked with °). Treatment with RNAse modifies the electrophoretic behaviour of HP1a and DDP1, confirming RNA-binding capability of HP1a and suggesting that interaction of DDP1 with HP1a is RNA-dependent. The apparent decrease of mobility observed after RNAse treatment of HP1-containing complexes could be the result of ribonucleic acid effect on electrophoretic mobility. RNA-containing proteic complexes migrate faster than the same complexes where RNA has been digested since RNA has a much higher charge/mass ratio than a protein.

### HP1a is mainly involved in RNA packaging and stability

**Figure 8:**
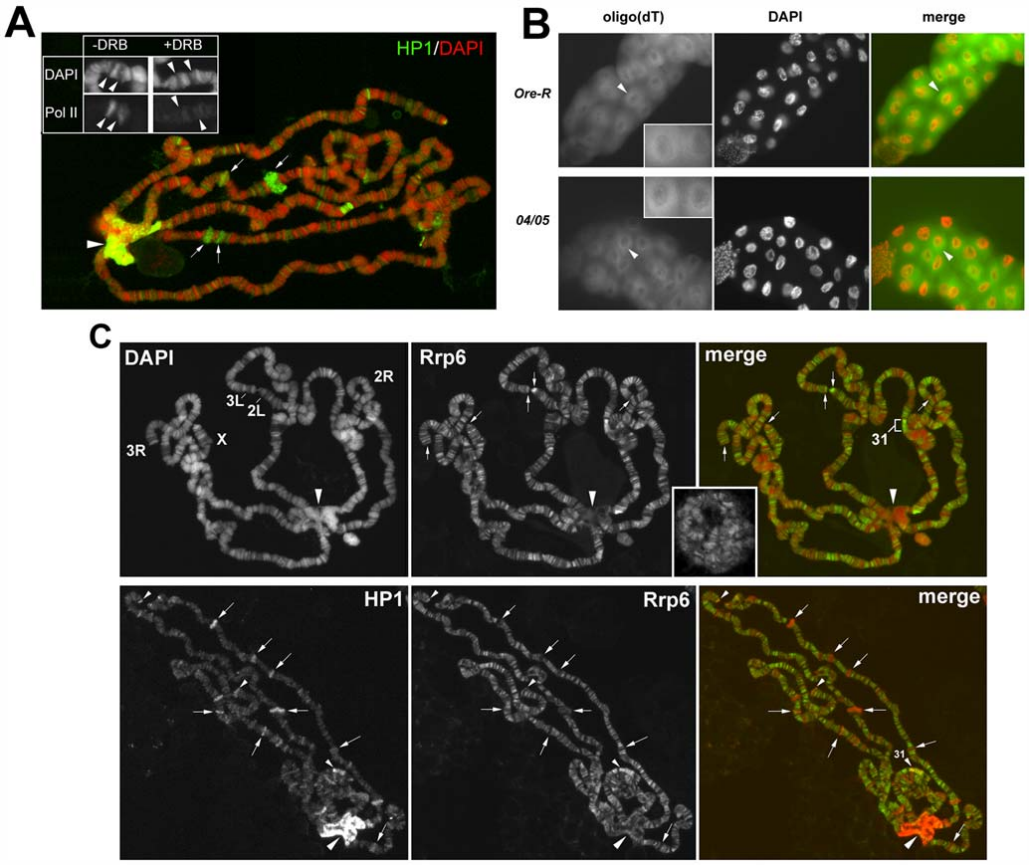
HP1a does not seem to be involved in transcript elongation, RNA export, or RNA surveillance. (A) Wild type heat-shocked polytene chromosomes treated with DRB. The treatment does not affect HP1a binding to the induced puffs (see arrows for examples). The absence of immunosignals in the insert shows that DRB does affects the Pol II Phospho Ser2 binding to heat-shocked puffs (arrowheads). (B) FISH detection of poly(A)^+^ RNA by a Cy3-labeled oligo(dT) probe in salivary glands from wild type and *Su(var)2–5^04^/Su(var)2–5^05^* HP1a mutant larvae. The tissues of both types of larvae show similar patterns (arrowheads). (C) Top, in polytene chromosomes of wild type larvae, the immunolocalization of the nuclear exosome component Rrp6. Note that the protein is present on many euchromatic sites including telomeres (arrows) and region 31, but absent on the heterochromatic chromocenter (big arrowhead). In the insert, a confocal microscopy image shows similar Rrp6 immunopattern also in not squashed polytenes. Bottom, the simultaneous immunolocalization of HP1a and Rrp6. Note that the two proteins colocalize mainly on telomeric regions and region 31 (small arrowheads); along the euchromatin there is little overlap (arrows indicate examples of regions that show positive HP1a and negative Rrp6 immunosignals).

Different classes of hnRNP proteins are involved in different aspects of RNA metabolism, such as transcript elongation, packaging and stability of mRNAs, RNA splicing, RNA surveillance and RNA export. In which of these functions is HP1a implicated? The cytological colocalization and the direct interaction of HP1a with active Pol II seem to be compatible with an involvement of HP1a in transcript elongation. We tested this hypothesis using the transcription inhibitor DRB on heat-shock induced puffs on polytene chromosomes. This treatment is known to precociously remove the elongation factors [34]. However, as shown in Figure 8A, HP1a remains at all the puffs even after the block of transcription. We also observed that the immunopattern of active Pol II is not affected on HP1a mutant polytene chromosomes (data not shown) indicating that RNA Pol II elongation is not disrupted in the absence of HP1a. Another suggested role for some hnRNPs is a shuttle function for the export of RNA from the nucleus to the cytoplasm [35],[36]. We analyzed the nuclear and cytoplasmic localization of poly(A)^+^ RNAs in wild type and HP1a mutants using a FISH detection of poly(A)^+^ RNAs in salivary glands from wild type and HP1a mutant larvae. The localization patterns of poly(A)^+^ RNAs observed in wild type are not changed in the absence of HP1a (Figure 8B). However, we cannot exclude specific changes in localization of HP1a-bound transcripts due to precocious release prior proper processing by hnRNPs or loading of stabilizing proteins. It is also unlikely that HP1a has a relevant role in RNA surveillance mechanisms. It is well known that mutations in nuclear exosome components increase the amount of transcripts [37]; HP1a mutations have the opposite effect. However, we further tested this suggestion by a simultaneous immunofluorescence staining of polytene chromosomes from wild type larvae, with C1A9 antibody and a specific antibody against the Rrp6 protein which is a nuclear component of the exosome [38]. We found little overlap of the two proteins. HP1a and Rrp6 colocalize at only a few euchromatic sites and at the telomeres (Figure 8C). Although in mammals the hnRNPA/B proteins seem to be important in pre-mRNA splicing, at least two lines of evidence exclude a relevant role for HP1a in this process. HP1a mutations have the same effects on genes with or without introns. Piacentini et al. (2003) have shown that HP1a mutations have a strong quantitative effect on *Hsp70* transcripts, though the *Hsp70* gene lacks introns and its transcripts do not require pre-mRNA splicing for their maturation. In addition, alterations in the levels of HRB87F have limited effects in alternative splicing in *Drosophila* [39].

**Figure 9:**
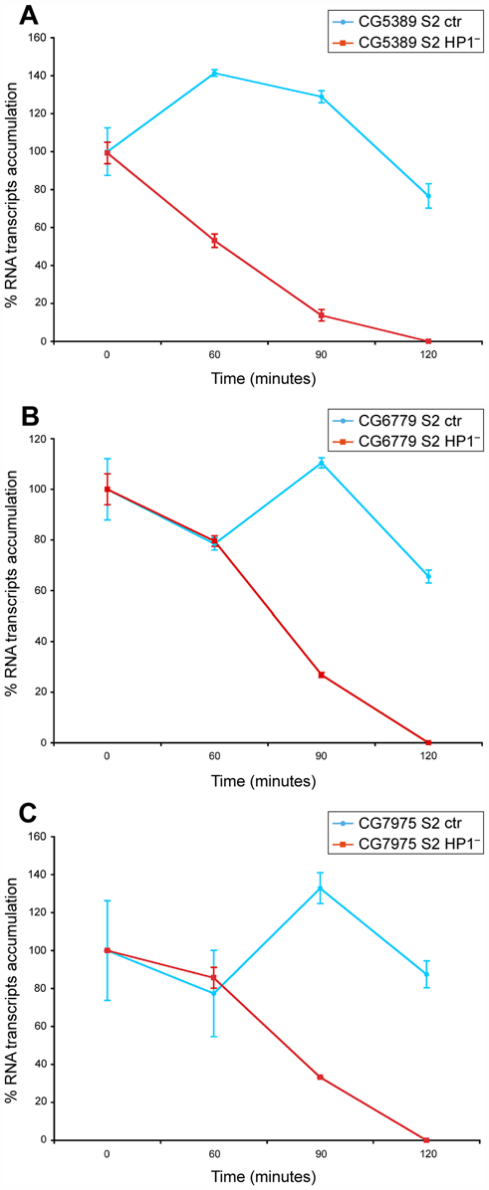
The HP1a target transcripts are less stable in cells lacking HP1a. Quantitative RT–PCR analysis of three HP1a target transcripts at different times after blockage of transcription by actinomicyn D treatment. The blue lines and the red lines respectively indicate the transcripts amount in S2 control cells and S2 cells interfered with HP1a dsRNA. Target transcripts correspond to genes: (A) CG5389, (B) CG6779, (C) CG7975. Note that at 120 minutes, the HP1a target transcripts are not detectable in HP1a depleted cells while in control cells their amount is only slightly decreased.

We think that the marked effect of HP1a mutations on the amount of RNA transcripts, together with its association with different types of hnRNPs which themselves apparently play a central role in RNA packaging and stability [33],[40], suggest that HP1a is also mainly involved in this function. In support of this idea, we found that after blocking transcription with actinomycin D, the HP1a target transcripts are less stable in S2 cells lacking HP1a compared to control cells (Figure 9).

### HRB87F and PEP, along with HP1a and DDP1, are involved in heterochromatin formation

**Figure 10:**
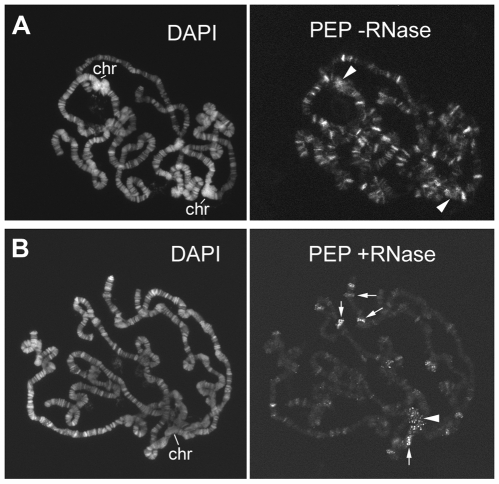
Modifications of PEP immunopattern on polytene chromosomes after RNase treatment. (A) PEP immunopattern on wild type polytene chromosomes. (B) PEP immunopattern on wild type polytene chromosomes after RNase treatment. Several immunosignals along euchromatin are removed while other immunosignals (arrows), including those on the chromocenter (arrowheads), become punctuated. Chr = chromocenter.

Though the immunofluorescence patterns of the hnRNP proteins are somewhat different, they seem to share common sites in the euchromatin of polytene chromosomes, and they are also present at the heterochromatic chromocenter. RNase treatment removes or modifies the euchromatic immunosignals of all these proteins, but it does not completely remove the heterochromatic immunosignals. For example, PEP is not completely removed from either the euchromatic sites or from the chromocenter, but the immunostaining loses its homogeneity and become punctuated (Figure 10). This change could be explained by the fact that PEP binds both DNA and RNA and that the RNase treatment removes only the protein bound to transcripts. On the other hand, the same RNase treatment seems to almost completely remove the euchromatic immunosignals and partially remove the heterochromatic immunosignals produced by the antibody against HRB87F (data not shown). In this case, it seems that the euchromatic localization of HRB87F depends exclusively on RNA, while its localization in different part of the heterochromatin depends on RNA or DNA.

**Figure 11:**
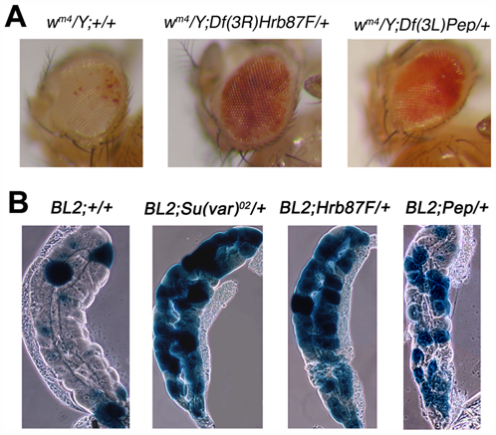
*Hrb87F* and *Pep* are dominant suppressors of position effect variegation (PEV). (A) In flies carrying the *In(1)w^m4^* rearrangement and deficiencies for either *Hrb87F* or *Pep*, the eyes are significantly more pigmented than in flies carrying only the chromosome inversion. *Hrb87F^KG02089^* and *Pep^KG00294^* mutations could not be tested because these mutations are caused by insertions containing a functionally wild type *white* gene. (B) Heat-shock *lac-Z* induction in salivary glands of Tp(3;Y)BL2 larvae which were also either wild type or heterozygous for *Su(var)2–5^02^*, *Hrb87F^KG02089^, Df(Hrb87F), Pep^KG00294^, Pep^EP(3)3357^, Pep^EP(3)0408^*, or *Df(Pep)*. The panel shows examples of staining patterns observed: in all heterozygous mutant larvae the glands stained more heavily than those of wild type larvae.

The heterochromatic convergence of these hnRNPs suggests that they, like HP1a, have a role in heterochromatin formation. A common test for the involvement of a gene in heterochromatin formation is to analyze its mutations for their effects on the heterochromatin-induced gene silencing called position effect variegation (PEV). The gene for DDP1 has already been shown to be a suppressor of PEV [25]. We tested *Hrb87F* and *Pep* mutations for their effects on the variegation of the *Stubble (Sb)* gene associated with *T(2;3)Sb^v^* [41]. In this translocation, the dominant neomorphic *Sb* mutation is relocated adjacent to the pericentromeric heterochromatin of the second chromosome. Flies carrying the translocation have a mosaic phenotype with *Sb* and wild type bristles. The normal bristle phenotype is due to the transcriptional repression of the dominant mutation. We crossed *T(2;3)Sb^v^* males to either *Hrb87F^KG02089^/TM3, Df(Hrb87F)/TM3, Pep^KG00294^/TM3, Df(Pep)/TM3* females. We compared the number of *Sb* and *Sb^+^* bristles in flies carrying the *T(2;3)Sb^v^* alone with flies who had the translocation and were also heterozygotes for either *Hrb87F^KG02089^, Df(Hrb87F), Pep^KG00294^, Df(Pep)* or *TM3* balancer chromosome. The results reported in Table 1 clearly show that mutations at *Hrb87F* and *Pep* are dominant suppressors of PEV: they significantly increase the frequency of *Sb* bristles with respect to the control. We also tested *Df(Hrb87F)* and *Df(Pep)* for their effects on the variegation of the *white (w)* gene associated with the *In(1)w^m4^* [42]. In this inversion, the *white* gene is transferred to a new position in the heterochromatin. In this location *white* undergoes a cis-heterochromatin inactivation that occurs in a certain proportion of the cells during development giving, for example in the eyes, a mosaic phenotype of mutant and wild-type areas. As shown in Figure 11A, the mutations dominantly suppress this type of PEV as well. We could not test the other *Hrb87F* and *Pep* mutations because they were induce by a P transposon insertion, which contains the *w^+^* gene. Instead we used *Tp(3;Y)BL2*, a Y chromosome rearrangement carrying the *Hsp70-lacZ* inducible transgene inserted into its centromeric region [43]. The heterochromatic location causes a variegation for the inducible *lac-Z* in salivary glands of larval males [44]. We constructed heterozygotes carrying either the *Su(var)2–5^02^*, *Hrb87F^KG02089^, Df(Hrb87F), Pep^KG00294^, Pep^EP(3)3357^, Pep^EP(3)0408^*, or *Df(Pep)* mutations and a balancer carrying an insertion of the GFP gene (to distinguish heterozygous larvae by the lack of GFP fluorescence). These females heterozygotes were crossed to *Tp(3;Y)BL2* males. As the examples in Figure 11B show, all the tested mutations dominantly suppress *lac-Z* variegation.

**Table 1:** Dominant effects of *Hrb87F* and *PEP* mutations on *Stubble* variegation of *T(2;3)Sb^v^*.

Mutant	No. of flies	total bristles^a^	% of *Sb* bristles	*P* b
*+* (control)	102	1428	16.8	
*TM3, Ser* c	262	3668	16.4	
*Hrb87F^KG02089^*	67	938	37.5	0,0001<*P*<0,005
*Df(3R)Hrb87F*	99	1386	37.0	0,0001<*P*<0,005
*Pep^KG00294^*	52	728	27.6	0,0001<*P*<0,005
*Df(3R)Pep^81k19^*	51	714	26.5	0,0001<*P*<0,005

a The bristles examined were the seven pairs of major dorsal bristles: posterior supraalars, anterior postalars, posterior dorsocentrals, anterior and posterior scutellars, and anterior and posterior sternopleurals (Sinclair et al., 1983).

b *P* values have been calculated using a χ2 contingency test.

c Total number of bristles counted in flies carrying the *T(2;3)Sbv* and the *TM3* balancer chromosome from all the crosses. Note that the proportion of the *Sb* bristles is similar to that of the control.

Since Hrb87F and PEP proteins appear to be required for heterochromatin-induced silencing, these proteins, like DDP1 and HP1a, are probably also involved in heterochromatin formation.

## Discussion

### HP1a upregulates gene expression by its association with RNA transcripts

Our data show that, among its multiple functions, HP1a is also involved in upregulation of many euchromatic genes at the postranscriptional level by an association of its chromodomain with the corresponding transcripts.

To identify HP1a targets, we performed a RIP-chip assay in S2 cells and we found that HP1a binds many RNA transcripts. Using a stringent cutoff (10% top rank), we identified target transcripts corresponding to about 100 genes. However, we think that the number of genes whose transcripts are affected by HP1a is probably much higher.

Although these transcripts were identified in cultured somatic cells, their genes correspond for the most part with HP1a immunosignals along the polytene chromosomes of larval salivary glands. The analysis of the expression of about 15% of these genes in S2 cells lacking HP1a and in HP1a mutant larvae showed a significant quantitative reduction in their transcripts, thus suggesting that HP1a is involved in their upregulation. For the first time we have been able to systematically identify the direct targets of HP1a in the euchromatin, and to determine HP1a's positive regulatory role on the corresponding genes.

### HP1a seems to be involved in RNA transcript packaging and stability by its interaction with hnRNP proteins

We found that HP1a binding sites overlap extensively with those of the DDP1, HRB87F and PEP proteins, and that it coimmunoprecipitates with these proteins. Though these proteins belong to different classes of hnRNPs, we found that they are part of a hnRNP sub-complex with an ordered assembly. They bind RNA transcripts in a hierarchical fashion beginning with DDP1 and ending with PEP.

In which aspect of RNA metabolism is HP1a involved? Our tests of the different possibilities lead us to conclude that the predominant role of HP1a may be the packaging and stability of RNA transcripts. This conclusion seems to be supported by the functional characteristics of the HP1a-interacting hnRNPs. For instance, A1 hnRNP is considered one of the best examples of a non specific RNA chaperone, probably required to prevent and resolve RNA misfolding, such as the formation of secondary structures that would be counterproductive to rapid processing of pre-mRNA [45],[46]. In conclusion, overall data indicate an involvement of HP1 in packaging and stability of RNA by binding to nascent transcripts at the transcription sites. We do not know yet if HP1-containing complexes also include processed RNAs.

### A novel role of hnRNP proteins in heterochromatin formation

The HP1a-containing hnRNP sub-complex also plays a role in heterochromatin formation. We found that the HRB87F and PEP proteins, like DDP1, are located on the heterochromatin, where they probably bind both RNA and single-stranded DNA, since they are partially removed from the chromocenter of polytene chromosomes after *in vivo* treatments with RNase. Importantly, we also found that these proteins, like DDP1, are dominant suppressors of heterochromatin-induced gene silencing. This strongly suggests that at least some hnRNP proteins have a novel and unexpected role in heterochromatin formation. A comparison of the heterochromatic immunopatterns of all these proteins reveals that DDP1, HRB87F and PEP are located in sub-regions of the chromocenter while HP1a is present on the entire chromocenter. Localization of each protein on polytene chromosomes of mutant larvae for each of the other genes also shows that DDP1 seems to be necessary for the recruitment of HRB87F and PEP proteins on the chromocenter. DDP1 and Hrb87F mutations only partially affect the HP1a heterochromatic pattern. It reasonable to think that DDP1, HRB87F and PEP bind some unknown non-coding RNA and/or single-stranded DNA. The partial modification of the HP1a immunopattern in DDP1 and HRB87F mutants implies that HP1a also binds a non-coding RNA in some heterochromatic sub-regions. Supporting this idea is our observation of a partial removal of the HP1a heterochromatic immunopattern after RNase treatment (unpublished data). The new role of HP1a in RNA processing suggests a possible additional contribution to the classical role of such protein in PEV. It is possible that genes inserted into or near heterochromatin could be transcribed more slowly, and enhancement of processing by HP1a could result in aberrant transcripts leading to post-transcriptional silencing. Reduced dosage of HP1a could shift the balance toward more productive transcripts, resulting in suppression of PEV.

It is evident that HP1a is a functionally multifaceted adaptor involved not only in heterochromatin formation, gene silencing and telomere capping, but also in the regulation of gene expression. What molecular mechanisms are responsible for the functional versatility of HP1a? Either HP1a possesses several modes of action, or HP1a always performs the same activity but with different partners in different contexts. In either case, we think that conformational changes due to post-translational modifications, generating a sort of an epigenetic sub-code, would permit the different interactions of HP1a in different contexts.

We propose that, regardless of the mechanism(s) of action, the main function of HP1a is nucleic acid compaction: HP1a's interaction with modified histones and specific hnRNP proteins, and perhaps some non coding RNA, produces a compaction of DNA that is the basis for heterochromatin formation and gene silencing, while HP1a interaction with RNA-packaging hnRNP proteins induces a compaction of RNA with the consequent stabilization that reinforces gene expression.

## Materials and Methods

### *Drosophila* strains

The *Ore-R, In(1)w^m4^*, *T(2;3)Sb^v^* and *T(3;Y)BL2* stocks used here have been kept in our laboratory for many years. The *Su(var)2–5* mutant strains were obtained from G. Reuter. *Su(var)2–5^05^* is a null mutation and *Su(var)2–5^04^* encodes a truncated HP1a protein that lacks part of the domain required for its nuclear localization; the protein is absent in mutant nuclei [8],[47]. *Su(var)2–5^02^* is a point mutation in the chromodomain [48]. The *ddp1* strain was obtained from F. Azorin. The mutant strain carrying a null *Hrb87F* mutation, called *Df(3R)Hrb87F*, was obtained from S. Haynes. The other *Drosophila* stocks mentioned were obtained from Bloomington and Szeged Stock Centers. Cultures were maintained at 24°C on standard cornmeal-sucrose-yeast-agar medium.

### Immunofluorescence

Indirect immunofluorescence on polytene chromosomes was done according to James et al. (1989). Salivary glands were dissected in Cohen and Gotchell medium G containing 0.5% Nonidet P-40 and incubated in formaldehyde fixative solution for 25 minutes. For DRB treatments, one gland of the pair was dissected in medium G, the other was incubated in medium G plus 140 µM DRB (5,6-Dichloro-l-β-D-ribofuranosylbenzimidazole). For ribonuclease digestion, dissected glands were incubated at room temperature with 50 µg/ml DNase-free RNaseA in medium G. The preparations were incubated with primary antibodies: goat anti-HP1a (1∶50) (Santa Cruz) or monoclonal mouse anti-HP1a C1A9, monoclonal mouse H5 (1∶50) to the Ser-2 phosphorilated CTD of RNA Pol II (Covance), mouse anti-DDP1 (1∶50), mouse anti-PEP (1∶2), mouse anti-HRB87F (1∶10) alone or in various pairwise combinations, overnight at 4°C in a humid chamber. The slides were washed in TBST (10 mM Tris-HCl, pH 7.15, 150 mM NaCl and 0.05% Tween 20) three times for 5 min and incubated with secondary antibodies (1∶100 dilution of FITC-conjugated donkey anti-mouse and 1∶400 dilution Cy3-conjugated rabbit anti-goat) (Jackson ImmunoResearch Laboratories) for 1 hour at room temperature in a humid chamber. Finally the slides were washed three times in TBST at 4°C, stained with 4,6-diamidino-2-phenilindole (DAPI) at 0.01 µg/ml, and mounted in antifading medium. Chromosome preparations were analyzed using a computer-controlled Eclipse epifluorescence microscope (model E1000, Nikon) equipped with a CCD camera (Coolsnap). The fluorescent signals, recorded separately as greyscale digital images, were pseudocolored and merged using Adobe Photoshop. For colocalization of HP1a with each protein, salivary glands from 10 larvae were prepared and about 3 good polytene nuclei of each larva were examined. The colocalization was performed by considering the fluorescent signals whose patterns were stably conserved among the different preparations. To determine the immunopatterns in the different mutants, same CCD camera exposure times were used (0.2 sec for FITC or Cy3 and 0.05 sec for DAPI). When required, the fluorescence of the signals was measured with the Adobe Photoshop program [49].

### Fluorescence in situ hybridization (FISH) with oligodT-Cy3

The FISH with oligodT-Cy3 was performed according to Herold et al., (2001) [50]. Salivary glands were dissected in physiological solution (0.7% NaCl) and fixed with 3.7% paraformaldehyde in PBS1X for 10 min. After fixation, salivary glands were washed in PBS1X, permeabilized for 10 min with PBS1X containing 0.5% Triton X-100 and washed again in PBS1X. To detect poly(A)^+^RNA, salivary glands were incubated for 30 min at 37°C in prehybridization buffer (2XSSC, 20% formamide, 0.2% BSA, 1 mg/mL of total yeast tRNA). For hybridization, the glands were transferred to a humidified chamber and incubated in 20 µl of hybridization buffer (prehybridization buffer plus 10% dextran sulfate) supplemented with 0.5 pmol/µl oligo(dT)50 fluorescently end-labeled with Cy3 molecules. The glands were hybridized for 3 h at 37°C and washed successively twice for 5 min in 2XSSC/20% formamide (at 42°C), 2XSSC (at 42°C), 1XSSC, and PBS. DNA was stained with DAPI and the slides were mounted in antifading medium.

### Histochemical localization of β-galactosidase in PEV analysis

The induction and localization of β-galactosidase on salivary glands was performed according to Lu et al., (1996) [44]. Heat-shock was done by incubating larvae for 45 min at 37°C, followed by a 1 h recovery at room temperature. Salivary glands were dissected in PBS, fixed in 5% formaldehyde for 15 min, washed with PBS, incubated in 0.2% X-gal (5-bromo-4-chloro-3-indolyl-p-D-galactopyranoside) assay buffer [44]. For each mutation, twenty pairs of salivary glands were analyzed.

### UV cross-linking assay

The assay was performed according to Hamann and Strätling (1998) [33]. Photoreactive ^32^P-labeled RNA probe (specific radioactivity 300 000 c.p.m./ng) was transcribed *in vitro* and purified by denaturing polyacrylamide gel electrophoresis in elution buffer (0.3 M NaAc pH 5.5, 0.1 mM EDTA, 0.2% SDS) in presence of phenol and 10 µg of yeast tRNA.

Binding reactions were set up in PCR-reaction tubes in the following order (total volume 50 µl): 5 µl 10X binding buffer [50 mM HEPES, pH 7.5, 15 mM MgCl_2_, 0.2 M KCl, 25% (v/v) glycerol, 5 mM dithiothreitol], different concentrations of recombinant HP1a protein, 3 µg yeast tRNA as non-specific competitor, and 50 ng radiolabeled RNA. Reactions were incubated at 25°C for 25 min. Samples were then irradiated in a UV Stratalinker using a total energy of 800 mJ/cm^2^ at 254 nm.

UV cross-linked samples were digested with 20 µg RNase A (Roche) and RNase V1 (Ambion) at 37°C for 20 min. After addition of 12.5 µl sample buffer [10% (w/v) SDS, 321.5 mM Tris-HCl, pH 7.5, 50% (v/v) glycerol, 700 mM 2-mercaptoethanol, 0.12% (w/v) bromophenol blue] and incubation at 95°C for 4 min, complexes were resolved on SDS-10% polyacrylamide gel, and dehydrated gels were exposed to X-ray film.

### Primer extension assay

The oligonucleotides Hsp70 R1 5′-TGCCCAGATCGATTCCAA-3′, and β tubulin 60D 5′-TAGCTGCTGCTGGATTTTCA-3′ antisense respectively of the 5′ UTR Hsp70Ab and β tubulin RNAs, were labeled for 30 min at 37°C with 25 µCi of [γ ^32^P]ATP using 10 units of polynucleotide kinase (Roche) in a 20 µl reaction volume. The samples containing labeled primers and immunoprecipitated RNAs were denatured at 95°C for 1 min, and then allowed to cool slowly to room temperature for the primer annealing. The primer extension reaction was performed at 43°C for one hour by adding 5 µl of the first-strand reaction mix (First-strand cDNA synthesis kit, Pharmacia), then terminated by precipitating with 2.5 vol of ethanol and 10% NaAcetate 3 M pH 5.5. The primer extension products were analyzed on a 6% DNA sequencing gel.

### Purification of HP1a protein constructs and electrophoretic mobility shift assay (EMSA)

HP1a constructs (1–206; 1–152; 1–95 and 152–206; 95–206) [21],[22] were cloned in *EcoRI/XhoI* sites of a pET-21a expression vector (Novagen) [15]. Corresponding recombinant proteins were produced by the Expressway Plus Epression System (Invitrogen), purified by the Ni-NTA purification System (Invitrogen) and eluted with 250 mM imidazol.

The gel mobility shift assay was done with a 400 bp fragment of *in vitro* transcribed Hsp70 RNA. For RNA binding ∼5 fmol of probe was incubated for 30 min at 25°C with different amounts of purified recombinant HP1a fragments in 10 µL reaction buffer containing 20% glycerol, 0.2 mM EDTA, 20 mM Tris pH 7.5, 1 mM MgCl_2_, 1 mM DTT, 150 mM NaCl and ribonuclease inhibitors. The reaction mixture, containing the protein-RNA complexes, was then loaded on a non-denaturating 8% polyacrylamide gel and run in 0.5% TBE at 150 V for 16 h at 4°C. The gel was dried at 80°C for 1 h and the radioactive bands were visualized by phosphor imaging in Typhoon scanner.

### Preparation of nuclear extracts for RIP-Chip

*D. melanogaster* Schneider's cells (S2) were prepared as previously described by Andrews and Faller (1991) [51]. In a typical preparation, 8×10^6^ cells (grown in serum-free Schneider medium; GIBCO-BRL) were pelleted and resuspended in 1.5 mL cold PBS1X; the cell suspension was then transferred to a microfuge tube. Cells were pelleted for 10 seconds and resuspended in 400 µL cold Buffer A (10 mM HEPES-KOH pH 7.9 at 4°C, 1.5 mM MgCl_2_, 10 mM KCl, 0.5 mM dithiothreitol, 0.2 mM PMSF, 0.2 U/µl RNasin). The cells were allowed to swell on ice for 10 minutes, and then vortexed for 10 seconds. Samples were centrifuged for 10 seconds, and the supernatant fraction was discarded. The pellet was resuspended in 100 µL of cold Buffer C (20 mM HEPES-KOH pH 7.9, 25% glycerol, 420 mM NaCl, 1.5 mM MgCl_2_, 0.2 mM EDTA, 0.5 mM dithiothreitol, 0.2 mM PMSF) and incubated on ice for 20 min for high-salt extraction. Cellular debris were removed by centrifugation for 2 min at 4°C and the supernatant fraction was stored at −70°C. All buffers were prepared from double-distilled autoclaved water that had been treated with 0.1% DEPC (Sigma).

### RNA immunoprecipitation and reverse transcription

For RNA immunopreciptation, the nuclear extract was incubated with 50 µg of monoclonal C1A9 anti-HP1a antibody at 4°C overnight under continuous gentle movement. One-hundred microliters of protein G-Sepharose (Sigma) suspension (50% packed Sepharose in Buffer C) was added and the incubation was continued overnight as described. The beads were pelleted by 2 min centrifugation at 240 g at 4°C; the pellet was briefly washed three times with 1 mL of IP Wash Solution (150 mM NaCl, 50 mM Tris pH 7.5, 0.5% NP40) and the Sepharose was transferred for elution into a fresh plastic tube and pelleted again. The supernatant was then completely removed. To elute the immunocomplexes for protein analysis, an aliquot of beads was suspended in 50 µL SDS-PAGE sample buffer and incubated for 10 min at 90°C; following centrifugation the supernatant was removed and used in Western blot for testing the presence of HP1a protein.

To elute the immunoprecipitated RNAs, the pelleted beads were boiled in 200 µL of DEPC water for 5 min, spun, and the supernatant recovered; 1 mL of Trizol (Invitrogen) was added to 200 µL of supernatant and mixed, followed by the addition of 200 µL of chloroform. This mixture was incubated at 4°C for 5 min and then centrifuged at 12000 g for 15 min; the RNAs in the aqueous phase were precipitated with half volume of isopropanol; after precipitation, the RNAs were resuspended in 10 µL of DEPC water. Contaminating DNA was digested with RNase-free DNase I (Sigma). The RNA purified from the previous step was used as a template to synthesize cDNA using oligo dT, random hexamers and SuperScript reverse transcriptase III (Invitrogen) according to the manufacturer's protocol.

### cDNA amplification and labeling

The cDNA was used as template for a two-step random PCR amplification [52]; in Round A, Sequenase is used to extend randomly annealed primers (Primer A) to generate templates for subsequent PCR; during Round B, the specific primer B is used to amplify the templates previously generated and finally round C consists of additional PCR cycles to incorporate the amino allyl dUTP nucleotide. About 25 ng of each cDNA sample was used for two 8 min extensions with 2.7 mM Round A primer (5′-GTT TCC CAG TCA CGA TCN NNN NNN NN-3′, N being a mixture of all four nucleotides with 60% A+T and 40% G+C) at 37°C with 267 U/ml Sequenase version 2.0 (usb). DNA was denatured at 94°C for 2 min and cooled to 10°C , and Sequenase 2.0 was added between extensions. The resulting products were used as template for 25 cycles of PCR using 1 U/100 µl *Taq* polymerase (*Platinum Taq* Invitrogen) and 10 mM Round B primer (5′-GTT TCC CAG TCA CGA TC-3′). Finally this DNA was used as template for 25 cycles of PCR to incorporate the amino allyl dUTP nucleotides to which the fluorescent dye may be attached [53]. To remove Tris buffer which interferes with the indirect coupling, the aminoallyl-cDNA samples were desalted by filtering through a Microcon −30 and then mixed with the succinimidyl esters of the Cy3 or Cy5 dyes (Amersham Biosciences) in 0.1 M sodium bicarbonate buffer (pH 9); the coupling reaction was incubated overnight in the dark at room temperature. Each dye-labeled sample was purified by AutoSeq MicroSpin G-50 columns (Amersham Biosciences) following the manufacturer's directions.

### Microarray hybridization and scanning

For hybridization, washing and scanning of arrays we followed the Canadian Drosophila Microarray Center protocols (www.flyarrays.com). In detail, the dried, labeled cDNA pellets were resuspended in 80 µl of DIG Easy Hyb (Roche) hybridization buffer containing 0.5 mg/ml yeast tRNA and 0.5 mg/ml salmon sperm DNA. Finally the DNA probe was denatured by incubation at 65°C for 10 min and, after a brief centrifugation to spin down the drops, the mixture was pipetted onto a microarray; a coverslip was applied and the slide was placed in a microarray hybridization chamber (BioRad) and incubated overnight at 37°C.

After hybridization, the slide was submerged in 0.01% SDS 1%SSC until the coverslip slid off the surface; the slide was washed in a solution of 1%SSC and shaken at 50°C for 10 min, then washed once more by shaking in 0.1%SSC. The slide was dried by centrifugation for 3 min at 550 rpm and scanned with an ScanArray Lite Microarray Scanner (Packard Bioscience) with laser intensities chosen to maximize signals while avoiding pixel saturation. ScanArray express software was used to quantify hybridization signals; bad spots were flagged automatically by the software and subsequently each slide was inspected manually. Since each gene is represented by two replica spots on the array, data were treated with GEPAS on-line tool (http://gepas.bioinfo.cnio.es) which averaged the two spots. The arrays we scanned are produced by the Canadian Drosophila Microarray Centre located at the University of Toronto (www.flyarrays.com). The 12k1 platform is a primarily cDNA-based glass microarray. The array features 11,018 Berkeley Drosophila Genome Project cDNAs, 297 NIH Testis cDNAs and 432 gene sequences that were amplified from genomic DNA. Approximately 10,500 unique genes are represented by the above, corresponding to roughly 78% of the predicted genes in *Drosophila melanogaster* (FlyBase annotation release 3.2).

### Quantitative Real-Time PCR

The quantitative real time PCR was performed according to Perrini et al., (2004) [15]. RNA samples from whole larvae or S2 cells were isolated by Trizol reagent (Invitrogen) according to the manufacturer's instructions. First strand cDNA was synthesized from 2 µg of total RNA using Omniscript RT kit (Qiagen). PCR primers were designed with Primer Express Software (Applied Biosystems). Reactions were set up in triplicate using the SYBR Green PCR Master Mix (Qiagen). Real time quantitative PCR was performed using an Applied Biosystems Prism 5700 Sequence Detector. The reaction mixtures were kept at 95°C for 15 min, followed by 40 cycles at 95°C for 15 s and 60°C for 1 min. Fluorescence output results were captured and analyzed using Gene Amp SDS Software, version 1.3 (Applied Biosystems), and the threshold cycle (Ct) was used for assessing relative levels of target transcripts versus RpL32 (or actin) transcripts.

### RNA interference

*D. melanogaster* S2 cells were cultured at 24°C in Shields and Sang M3 medium (Sigma) supplemented with 10% heat-inactivated fetal bovine serum (FBS, Invitrogen).

dsRNA against HP1 was syntetized by *in vitro* transcription from PCR products with T7 promoters on both ends of the amplicons, using the Megascript RNAi kit (Ambion).

1×10^6^ cells were plated in 1 ml of serum-free medium in a well of a six-well culture dish (Sarstedt). Each culture was inoculated with 15 µg of dsRNA. After a 1 h incubation at 24°C, 2 ml of medium supplemented with 15% FBS were added to each culture. Control cultures were prepared in the same way but without addition of dsRNA. Both RNA-treated and control cells were grown for 72 h at 25°C, and then processed for total RNA extraction. The RNAi experiments were repeated two times to confirm the reproducibility of the observations.

### Actinomycin D treatments

For analysis of RNA transcripts turnover, S2 cells were treated with 10 µg/ml actinomycin D (Sigma) for 0, 60, 90 and 120 minutes. Total RNA was isolated by Trizol (Invitrogen), reverse transcribed by Omniscript RT kit (Qiagen) and analyzed by real time quantitative PCR.

### Western blot of coimmunoprecipitated hnRNPs

Coimmunoprecipitation was performed according to Risau et al. (1983) [54] using 50 µg of monoclonal C1A9 antibody for 1 ml of hnRNP extract. The immunocomplexes, fractionated by 10% SDS-PAGE, were electroblotted onto Immobilion-P polyvinyldifluoride membranes (Millipore) in a buffer containing 10 mM 3-cyclohexylamino-1-propanesulfonic acid (Sigma-Aldrich) pH 11 and 20% methanol, in a semi-dry transfer apparatus (Amersham Biosciences). The filter was blocked with 0.2% I Block (Tropix) for AP detection in PBS/0.1% Tween 20 (PBST). After blocking, proteins were probed with antibody against HP1a (1∶500), DDP1 (1∶3,000), HRB87F (1∶100), PEP (1∶100) and detected with a 1∶5,000 dilution of goat anti–mouse or anti-rabbit conjugated to alkaline phosphatase. The AP detection kit was purchased from Roche.

### Immunopurification of HP1a complexes

S2 cells were transfected with pAWF plasmid coding either for HP1a or GFP protein, both FLAG-tagged. Aliquots of 3×10^6^ cells were plated into 75 cm^2^ flasks and, after overnight incubation in M3 insect medium (Sigma), were transfected with 9 µg of plasmid DNA for 5 h using Cellfectin II reagent (Invitrogen) following manufacture's procedure. Cells were grown for further 48 h and then harvested by centrifugation. Nuclei were obtained by cell lysis in hypotonic buffer (10 mM Hepes pH 7.4, 10 mM KCl, 5 mM MgCl_2_, 0.5 mM EGTA, 10% glycerol, 10 mM sodium glycerophosphate, 0.4 mM PMSF, 0.2 mM sodium Na_3_VO_4_, 30 mM NaF ). Aliquots of 3×10^7^ cells, transfected with either HP1a or control plasmid, were incubated on ice for 20 minutes in hypotonic buffer and then centrifuged at 1500 g for 10 minutes. Nuclei were washed by resuspension in hypotonic buffer and centrifugation. Nuclear extracts, corresponding to nucleoplasm, were obtained by incubation of nuclei in lysis buffer (50 mM Tris-HCl, 150 mM NaCl, 0,5 mM EDTA, 10% glycerol, 0.5% NP40, 0.5 mM DTT, 0.4 mM PMSF, 0.2 mM Na_3_VO_4_) for 20 minutes at 4°C followed by centrifugation at 15000 g for 20 minutes. Supernatants were further processed for immunopurification with anti-FLAG monoclonal antibodies. To obtain total nuclear lysate, nuclei were resuspended in lysis buffer and chromatin sheared by sonication (six times for a 20 second pulse at 80% power setting using a Braun Biotec-Sartorius ultrasonicator equipped with a 2 mm tip). Lysates were processed for immunopurification with anti-FLAG antibodies.

Nuclear extracts and nuclear lysates obtained as above were immunopurified by adding 36 µg of anti-FLAG antibodies (Sigma) and 200 µl of protein-G-Dynabeads (Invitogen) followed by overnight incubation at 4°C. Immunocomplexes were isolated onto a magnetic support, washed three times in lysis buffer and eluted by overnight incubation at 4°C with 200 µl of 3x FLAG peptide (Sigma) 300 µg/ml in lysis buffer.

Fractions obtained by immunopurification were resolved either by 10% SDS-PAGE or 6% native-PAGE. Native gel electrophoresis was performed using the discontinuous Laemmly buffer system without SDS, running gels at 100 V for 3 hours. Mobility of nuclear complexes was tested before and after RNAse treatment by adding 1 µg of RNAseA to each sample before separation followed by 1 h incubation at room temperature. Gels were transferred onto PVDF membranes and subjected to western blotting with anti-FLAG (1∶1000), anti-DDP1 (1∶3,000), anti-HRB87F (1∶100) and anti-PEP (1∶100) antibodies. Membranes were blocked overnight with 3% non-fat dry milk in TBS containing 0.1% Tween-20 (TBST) followed by incubation with primary antibodies. Membranes were probed with a 1∶5,000 dilution of anti-mouse or anti-rabbit antibodies conjugated to alkaline phosphatase and signals were detected using the CPD-Star chemiluminescent kit (Roche) and X-ray films (Amersham).

### Data availability

Microarray data are available in the ArrayExpress database, http://www.ebi.ac.uk/miamexpress, under accession number E-MEXP-1556 (ChIP-chip records).

## Supporting Information

Figure S1 Plots of Log2 HP1a-Dam/Dam ratio for coding-region probes of six genes present on chromosome 2 which show high IP rankings for HP1a RNA binding. Probes are ordered from 5′ to 3′ and the direction of transcription is shown by the arrow.
(5.43 MB TIF)

Figure S2 HP1a associates and colocalizes on polytene chromosomes with Pol II, DDP1, PEP, and HRB87F hnRNP proteins. Wild-type polytene chromosomes simultaneously immunostained with an anti-HP1a antibody and an antibody against: (A) Pol II (B) DDP1; (C) PEP and (D) HRB87F. Note the extensive colocalization of HP1a with all the proteins along the euchromatic arms (small arrows) and on the chromocenter (arrowheads).
(3.48 MB TIF)

Figure S3 Hierarchical dependence of HP1a, DDP1, HRB87F, and PEP in their assembly on RNA transcripts. Immunopatterns of each protein on polytene chromosomes of wild-type larvae (top row) and larvae mutant for the genes encoding each of the other proteins. The pictures inside the red frame indicate the abnormal immunopattern of a protein in a mutation affecting another protein. The pictures inside the blue frame indicate the absence of immunosignals of each protein in the mutant of its own gene, except for the *Su(var)2–5^02^* mutant where weak immunosignals are visibile on the chromocenter, on telomeres and very few euchromatic sites. The rest of the pictures are of immunopatterns not affected by the mutations. Note that in DDP1 mutants, immunopatterns of all the other proteins are abnormal. In *Hrb87F* mutants, the HP1a and PEP immunopatterns are abnormal while the DDP1 immunopattern is unaltered. In HP1a mutants only the PEP immunopattern is abnormal, whereas in PEP mutants the immunopatterns of all other proteins are normal.
(3.49 MB TIF)

Table S1 Data from 4 independent RIP-Chip experiments. For each experiment, data were ordered by rank and the median rank was calculated for each cDNA clone.
(2.04 MB XLS)

Table S2 Median percentile rank (MPR) between IP and input for the cDNA clones within the 90th percentile. The table also reports the results of two independent (mock) experiments done with the identical procedure but without the antibody against HP1.
(0.03 MB XLS)

Table S3 Co-localization of HP1 target genes and HP1 immunosignals along polytene chromosomes (green).
(0.03 MB XLS)

Table S4 The 200 most abundant transcripts in the input RNA and their percentile ranking in HP1 IP. Only 6 transcripts (indicated by yellow) are included in the list of the best HP1 binders (IP ranking>0.9).
(0.04 MB XLS)

Table S5 Log2 HP1-Dam/Dam ratios for all probes representing the coding regions of 21 genes of chromosome 2 which show high MP ranks for HP1 RNA binding. Average values are indicated.
(0.49 MB XLS)
