# Supplementary figures and images for: Heterochromatin Protein 1 (HP1a) Positively Regulates Euchromatic Gene Expression through RNA Transcript Association and Interaction with hnRNPs in Drosophila
Source: PLoS Genet. 2009 Oct 2;5(10):e1000670. doi: 10.1371/journal.pgen.1000670 (PMC2743825; doi:10.1371/journal.pgen.1000670)

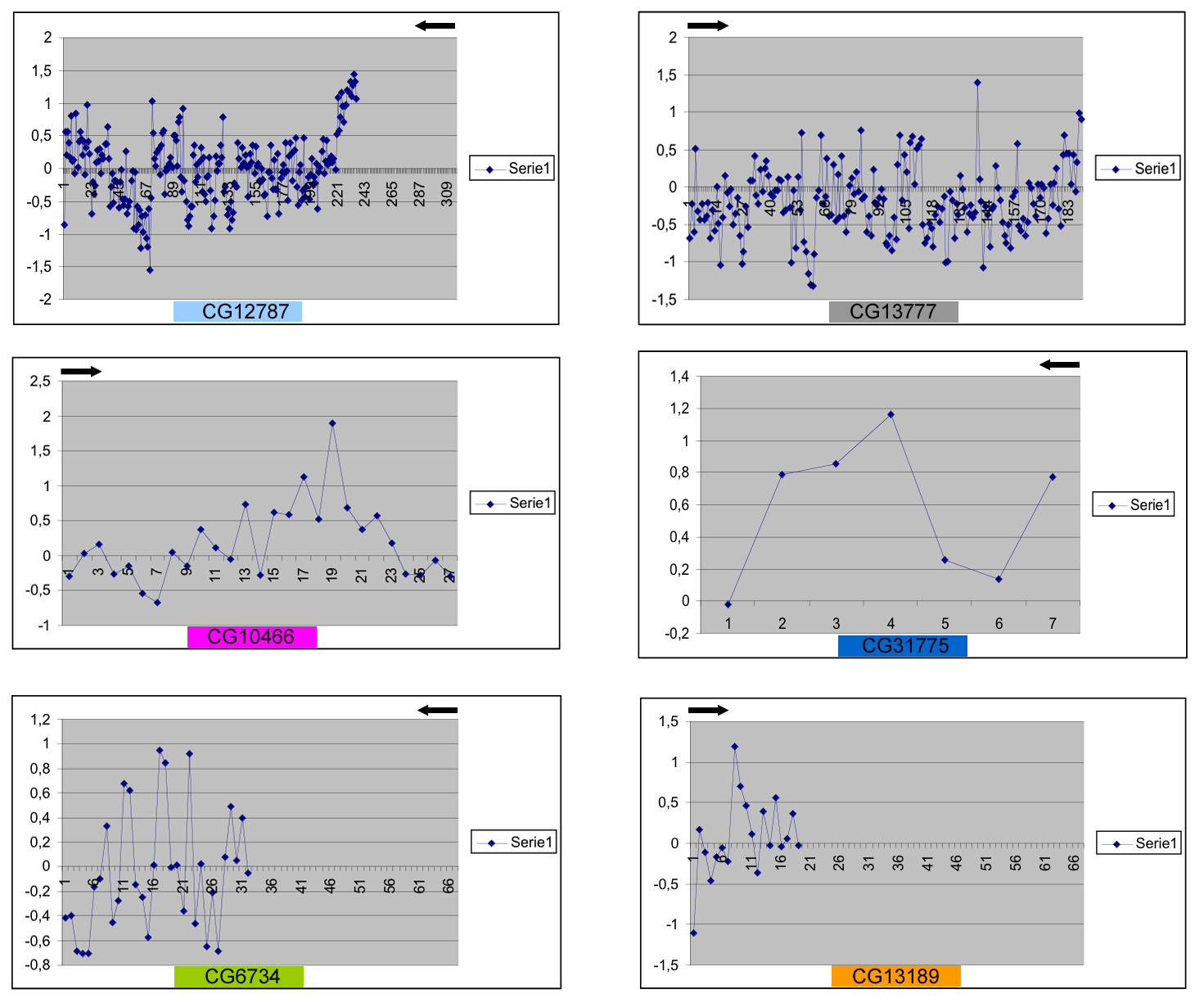

Supplement: Figure S1 — Plots of Log2 HP1a-Dam/Dam ratio for coding-region probes of six genes present on chromosome 2 which show high IP rankings for HP1a RNA binding. Probes are ordered from 5′ to 3′ and the direction of transcription is shown by the arrow. (5.43 MB TIF) [file pgen.1000670.s001.tif]

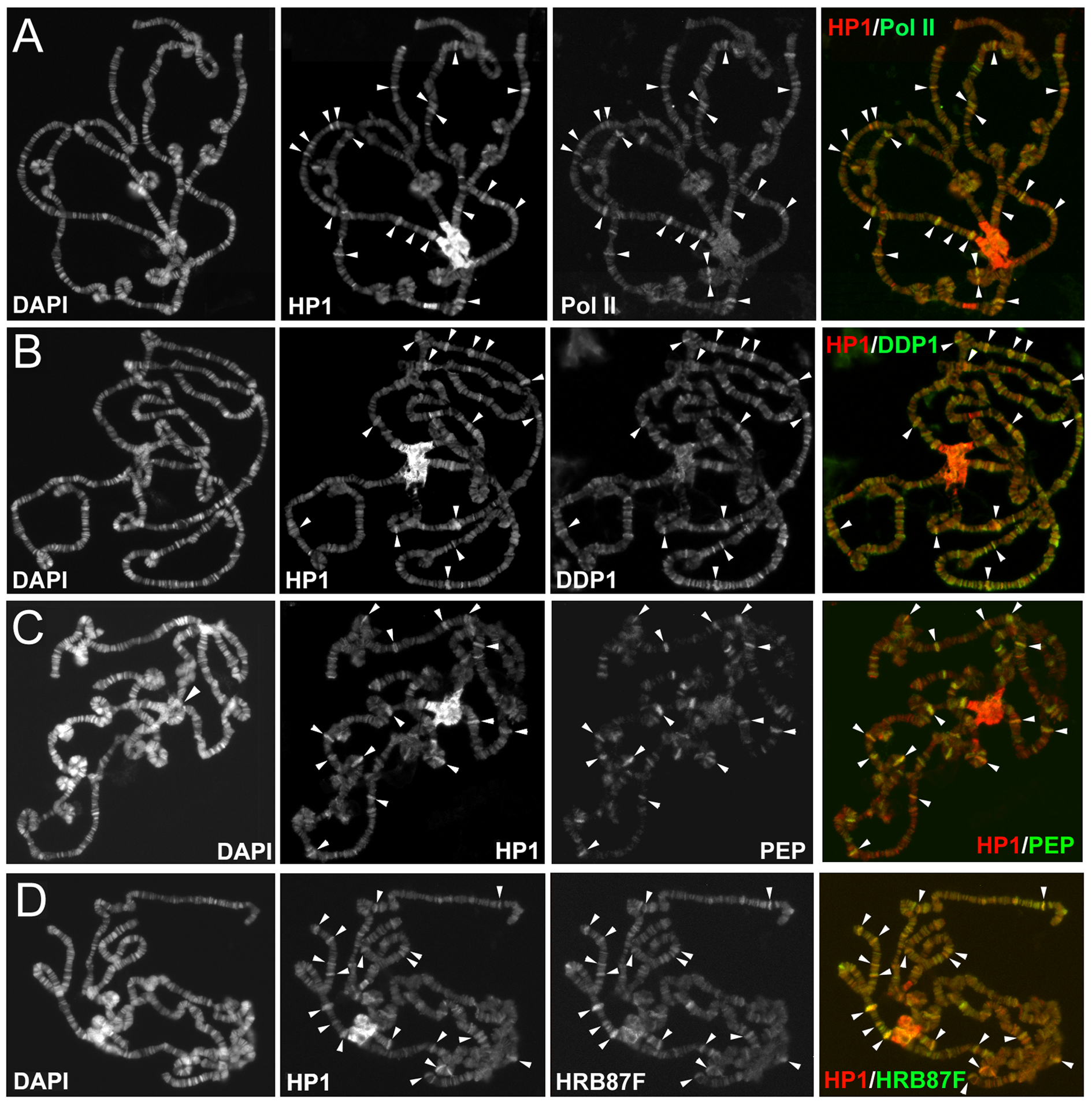

Supplement: Figure S2 — HP1a associates and colocalizes on polytene chromosomes with Pol II, DDP1, PEP, and HRB87F hnRNP proteins. Wild-type polytene chromosomes simultaneously immunostained with an anti-HP1a antibody and an antibody against: (A) Pol II (B) DDP1; (C) PEP and (D) HRB87F. Note the extensive colocalization of HP1a with all the proteins along the euchromatic arms (small arrows) and on the chromocenter (arrowheads). (3.48 MB TIF) [file pgen.1000670.s002.tif]

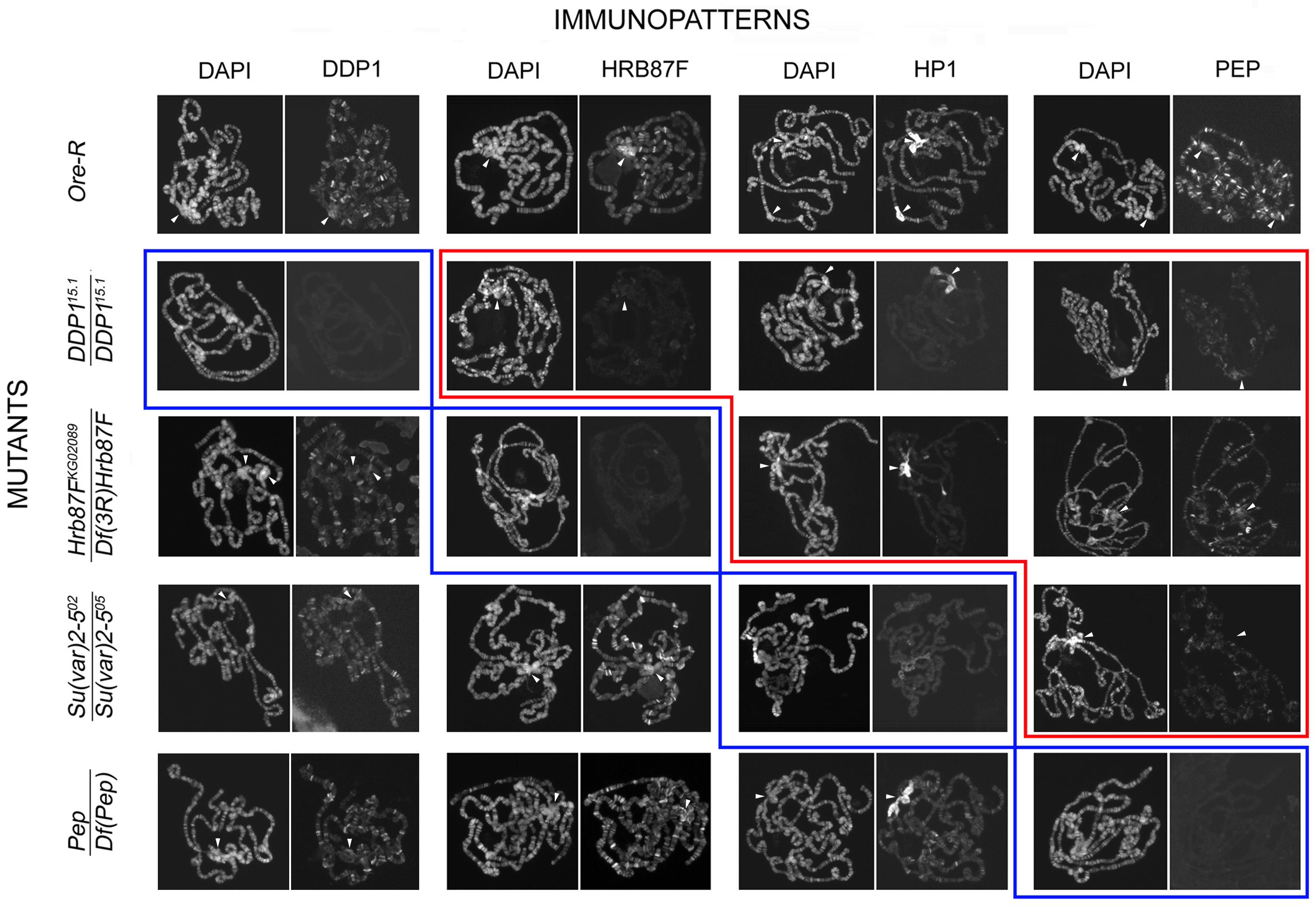

Supplement: Figure S3 — Hierarchical dependence of HP1a, DDP1, HRB87F, and PEP in their assembly on RNA transcripts. Immunopatterns of each protein on polytene chromosomes of wild-type larvae (top row) and larvae mutant for the genes encoding each of the other proteins. The pictures inside the red frame indicate the abnormal immunopattern of a protein in a mutation affecting another protein. The pictures inside the blue frame indicate the absence of immunosignals of each protein in the mutant of its own gene, except for the Su(var)2–502 mutant where weak immunosignals are visibile on the chromocenter, on telomeres and very few euchromatic sites. The rest of the pictures are of immunopatterns not affected by the mutations. Note that in DDP1 mutants, immunopatterns of all the other proteins are abnormal. In Hrb87F mutants, the HP1a and PEP immunopatterns are abnormal while the DDP1 immunopattern is unaltered. In HP1a mutants only the PEP immunopattern is abnormal, whereas in PEP mutants the immunopatterns of all other proteins are normal. (3.49 MB TIF) [file pgen.1000670.s003.tif]
